# Exploring Perspectives of Patients With Cancer on Implementing Electronic Patient-Reported Outcome Measures to Enhance Patient-Centered Care: Qualitative Study

**DOI:** 10.2196/79144

**Published:** 2025-11-25

**Authors:** Terese Solvoll Skåre, Tonje Lundeby, Jo-Åsmund Lund, Elias David Lundereng, Stein Kaasa, Nienke de Glas, Karianne Røssummoen Øyen, Kristin Vassbotn Guldhav, May Helen Midtbust

**Affiliations:** 1 Department of Health Sciences Ålesund, Faculty of Medicine and Health Sciences, Norwegian University of Science and Technology, NTNU Ålesund, Postbox 1517, Ålesund, 6025, Norway, 47 41213973; 2 Department of Oncology, Regional Advisory Unit for Palliative Care, Oslo University Hospital, Oslo, Norway; 3 Department of Oncology, European Palliative Care Research Centre (PRC), Oslo University Hospital, Oslo, Norway; 4 Clinic for Cancer Treatment and Rehabilitation, Møre og Romsdal Hospital Trust, Ålesund, Norway; 5 Norwegian Centre for Clinical Cancer Research, MATRIX, Oslo, Norway; 6 Department of Oncology, Helse Førde, Førde, Norway

**Keywords:** patient perspective, patient engagement, qualitative research, ePROMs, digital health solutions, eHealth, neoplasm, implementation science, patient-centered care, cancer care, electronic patient-reported outcome measures

## Abstract

**Background:**

Systematic symptom management is a crucial component in patient-centered cancer care. Despite the development of numerous electronic patient-reported outcome measure (ePROM) tools, integrating these tools into clinical practice remains challenging. Engaging key stakeholders, including patients, in the development of ePROM tools is pivotal to fostering the adoption of such tools. As part of an innovation and implementation study aimed at enhancing efficiency and patient-centered care (PCC) through the development of digital PCC pathways, we explored the perspectives of patients with cancer on current clinical practice regarding symptom management and PCC, as well as their needs and preferences related to ePROMs.

**Objective:**

This study aims to explore the perspectives of patients with cancer on PCC and symptom management, including their experience with current clinical practice and their views on how ePROMs might enhance patient-centered follow-up.

**Methods:**

A 2-stage qualitative design was used. In stage 1, semistructured individual interviews were conducted to gain an in-depth understanding of patients’ experiences with current clinical practice, including perceived challenges and unmet needs. Stage 2 involved structured interviews to further explore patients’ perspectives on the potential role of ePROMs in enhancing patient-centered follow-up.

**Results:**

A total of 10 patients were included in the study, participating in either or both stages. Two main themes were developed through a reflexive thematic analysis process: (1) symptom management in the shadow of disease-centered care, and (2) ePROMs: bridging holistic care and disease management. Theme 1 highlighted how patients made sense of symptom management within a health care context primarily focused on disease treatment and progression. Their narratives revealed that biomedical concerns often dominated clinical encounters, while patients’ broader lived experiences and symptom-related needs were marginalized. Patients shared an understanding that it was their own responsibility to redirect the focus of clinical consultations toward symptoms. While they generally expressed satisfaction with the care received, they also described a sense of unmet needs that remained unaddressed. The second theme explored how patients made sense of the potential role of an ePROM tool in supporting more patient-centered cancer care. Their accounts revealed both perceived barriers and facilitators to its use, shaped by the expectations and needs that contrasted with current clinical practices. Central to this was a belief, emerging through engagement with the conceptual tool’s functionalities, that it could enable a more holistic approach to care, extending beyond physical symptom to encompass the lived experience of cancer.

**Conclusions:**

Patients often felt personally responsible for ensuring that their symptoms were addressed, indicating shortcomings in follow-up and communication. ePROMs were identified as a promising tool to strengthen PCC by amplifying patient voices and enabling more holistic and responsive follow-up. Integrating ePROMs into routine care may improve symptom visibility, foster shared understanding between patients and health care professionals, and support more equitable care delivery.

## Introduction

Patient-centered care (PCC) has emerged as a critical component of high-quality cancer care. PCC is an approach to health care that prioritizes individual patients’ preferences, needs, and values, ensuring active involvement in all treatment and care decisions [1]. Previous research has shown that PCC improves patient well-being, satisfaction with and quality of care, self-efficacy, and trust toward health care professionals (HCPs) [2,3]. Despite extensive knowledge about the benefits of adopting a PCC approach, cancer care remains predominantly tumor-centered, with a primary focus on diagnostics and treatment [4,5]. This narrow focus often limits attention to the broader needs and experiences of the patient.

A central component of PCC is the systematic assessment of symptoms. Effective symptom management can enhance quality of life (QoL), facilitate early symptom detection, and may even contribute to improved overall survival [6]. Traditional cancer care, however, has primarily focused on curing the disease or controlling its progression, often at the expense of recognizing and addressing the broader symptom burden experienced by patients. Moreover, previous research suggests that symptoms are frequently undetected or underestimated by HCPs [7,8]. Discrepancies between clinician-reported and patient-reported symptoms have also been identified [9,10], alongside a tendency for patients to underreport their symptoms [11]. Therefore, strengthening PCC by prioritizing symptom assessment alongside conventional clinical treatment is of vital importance, as it facilitates a more holistic and patient-centered approach to cancer care. Actively involving patients and implementing systematic symptom management are, thus, essential strategies for achieving PCC in oncology.

Patient-reported outcome measures (PROMs) enable the direct collection of data regarding patients’ health status, including symptoms, QoL, and nutritional status [12]. Prior research indicates that PROMs enhance patient empowerment by fostering self-reflection, supporting individualized treatment, and facilitating shared decision-making [13]. When used in clinical practice, PROMs can bolster self-management and satisfaction with care and facilitate more effective patient-clinician communication [14-16]. In addition, PROMs can help identify supportive care needs and address sensitive or unexpected concerns [17-19]. Recent digital advancements have facilitated the development of numerous electronic patient-reported outcome measures (ePROMs), which have the potential to enable more efficient and accurate data collection from patients [20]. ePROMs can enhance data integrity, demonstrate cost-effectiveness, reduce administrative workload, and facilitate comparable or expedited completion times [21,22]. In addition, patients report high acceptability and satisfaction when using ePROMs, both in routine care and research settings [14,23].

Although the use of ePROMs in cancer care is increasing, there remains limited knowledge about how patients’ perspectives on PCC and symptom management can inform the development of these tools. Further research is needed to understand how ePROMs can be designed to reflect patients’ values and needs and to support more patient-centered follow-up in clinical practice. Moreover, understanding how ePROMs are implemented and used in clinical settings is essential for ensuring the development of tools that contribute meaningfully to the delivery of PCC in oncology. Therefore, this study aims to explore the perspectives of patients with cancer on PCC and symptom management, including their experiences with current clinical practice and their views on how ePROMs might enhance patient-centered follow-up. The insights gained will inform the development and implementation of ePROMs that are relevant, feasible, and meaningful in real-world clinical settings.

The aim was addressed through the following research questions: (1) What are the experiences of patients with cancer regarding symptom management and PCC in outpatient cancer treatment and care? (2) What are the perspectives of patients with cancer on the perceived usefulness of ePROM tools in enhancing PCC, and what factors influence their willingness to use such tools?

## Methods

### Design

A 2-stage, qualitative design was adopted [24]. In stage 1, semistructured individual interviews were conducted to gain an in-depth understanding of patients’ experiences with current clinical practice, including perceived challenges and unmet needs. In stage 2, structured interviews were carried out to further explore patients’ views on the potential role of ePROMs in enhancing patient-centered follow-up. This approach enabled a nuanced exploration of both experiential and evaluative dimensions [25,26], offering valuable insights into how ePROMs might be designed and implemented to more closely align with patients’ values, needs, and expectations.

### Setting

The study presented in this paper was conducted as part of the Norwegian MyPath-MATRIX project. Details of the main project have been published elsewhere [5,27]. In short, this project is a subproject of the MyPath-EU project, a multicenter, international implementation study funded by the European Commission [28,29]. The MyPath-MATRIX project aims to enhance PCC by developing and implementing PCC pathways supported by health and information technology (digital PCC pathways) at 3 oncology outpatient clinics in Norway [27]. One part of the care pathways is to assess patients’ symptoms and functioning through an ePROM tool.

A pivotal component of the digital PCC pathways is the systematic collection of information from patients regarding 6 core areas: pain, fatigue, nutrition, physical function, social function, and psychological distress [28]. Building on the previously used computer-based symptom assessment tool, Eir software (NTNU and St. Olavs Hospital) [30,31], the MyPath ePROM tool is currently under development to support personalized follow-up in cancer care. The tool is designed to assess patients’ well-being, symptoms, and functioning, including nutrition and psychological aspects. It uses an adaptive format: based on patients’ initial responses, it generates tailored follow-up questions to ensure a comprehensive and personalized assessment, thereby facilitating targeted interventions and support [29]. In this study, participants were presented with a conceptual version of the MyPath ePROM tool during the second interview stage. The tool was introduced using a PowerPoint presentation containing screenshots that illustrated the tool’s interface, example questions, and response options. This allowed participants to visualize how the tool might function in practice and to provide feedback on its content, design, and perceived usefulness in supporting person-centered follow-up in cancer care.

### Recruitment

All participants were recruited using a purposeful sampling strategy [24] based on specific inclusion and exclusion criteria (Textbox 1). Six oncologists from 2 oncology departments in Norway (4/2) were asked to identify patients who met the eligibility criteria. Their role was limited to distributing information sheets to these patients and asking whether they would be willing to be contacted by the researcher (TSS). The oncologists did not participate in the recruitment process beyond this initial step.

Textbox 1. **Inclusion and exclusion criteria for adult patients with cancer participating in a qualitative study conducted at 2 Norwegian hospitals.**

**Inclusion criteria**

- Age: ≥18 years
- Gender: All
- Medical care: Undergoing active chemotherapy or having one of the following cancer diagnoses: gastrointestinal or urologic
- Scope of treatment: Curative and palliative
- Language: Norwegian, spoken and written
- Consent: Able to provide informed consent

**Exclusion criteria**

- Patients who, due to their health conditions, were deemed too frail or ill to participate in an approximately 60-minute interview

**Figure 1.**
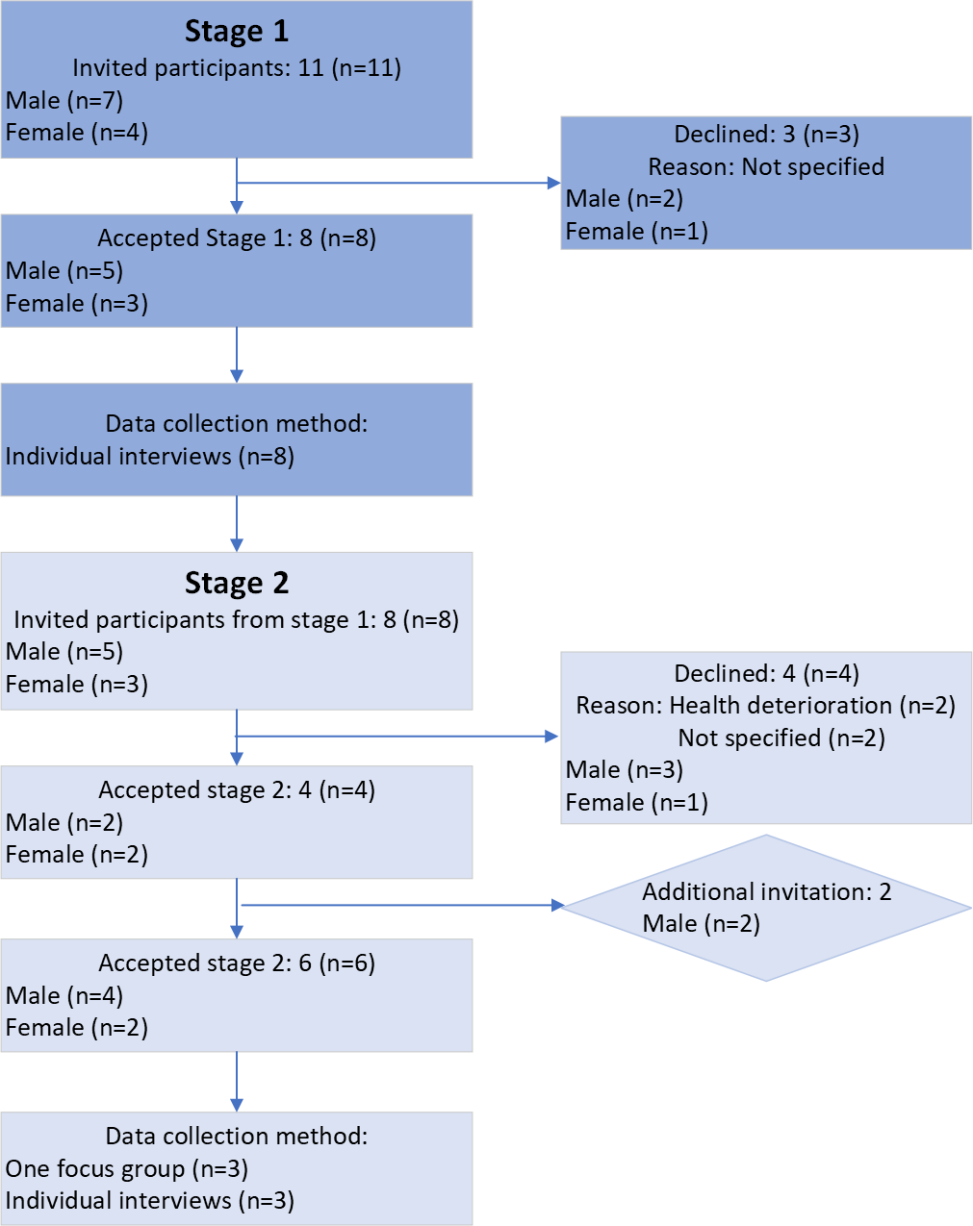
Flowchart of participant inclusion and progression in a 2-stage qualitative study. The study was conducted among adult patients with cancer receiving curative or palliative treatment at 2 Norwegian hospitals between February and September 2024. Data were collected in 2 stages through individual interviews and 1 focus group. The figure illustrates participant inclusion, gender distribution, declined participation, and allocation to stage 1 and stage 2.

In stage 1, 11 patients agreed to be approached for inclusion in the study. Of these, 8 agreed to take part. In stage 2, the 8 participants from stage 1 were invited to continue. Four consented to further participation, while the remaining 4 declined. To ensure sufficient data for stage 2, an additional 2 participants were recruited, resulting in a total of 6 participants in the second stage. In total, 13 patients consented to be contacted, and 10 patients ultimately participated in at least 1 stage of the study (Figure 1). Among these, 3 were women and 7 were men. The mean age was 65 years, ranging from 48 to 73 years.

### Data Collection

Stage 1 focused on exploring patients’ experiences with symptom management and PCC. Eight individual interviews were conducted using a semistructured interview guide. The interviews lasted between 33 and 86 minutes (mean duration 56 minutes). The guide was designed to allow flexibility in questioning, enabling the interviewer to adapt follow-up prompts based on each participant’s responses [32].

The interview guide comprised three overarching themes, each explored through open-ended questions: (1) patients’ experiences with PCC, (2) current practices in symptom follow-up, and (3) perspectives on the use of a digital tool to support symptom management and PCC. Follow-up questions such as, “Could you give an example of what you just mentioned?,” or “Could you elaborate on what you mean by...?” were used to encourage deeper reflection and elaboration. This approach facilitated the generation of rich, nuanced data on participants’ lived experiences and expectations [33]. The guide, including key themes and primary questions, is available in Multimedia Appendix 1.

In stage 2, data collection was initially planned to include focus group (FG) discussions; however, due to participants’ reluctance to attend sessions outside of their already scheduled clinical appointments, only 1 FG was conducted (n=3). In addition, 3 individual interviews were carried out. The FG lasted 106 minutes, while the individual interviews ranged from 56 to 71 minutes. In stage 2, patients were presented with a conceptual version of the ePROM tool, comprising preliminary visual representations of proposed content and functionalities. This version was not intended for clinical implementation but served as a prototype to facilitate discussion and elicit feedback on design elements, usability, and relevance. The materials were used to prompt reflection on participants’ needs and preferences and to assess the perceived value and usefulness of such a tool in their care context.

The interview guide used in stage 2 was structured, in contrast to the semistructured format used in stage 1. It was designed to align closely with the visual materials presented during the interviews, enabling a focused exploration of participants’ responses to specific features and concepts. Topics included perceived needs and preferences for digital symptom–reporting tools, as well as feedback on the design, content, and functionality of the proposed ePROM interface.

**Figure 2.**
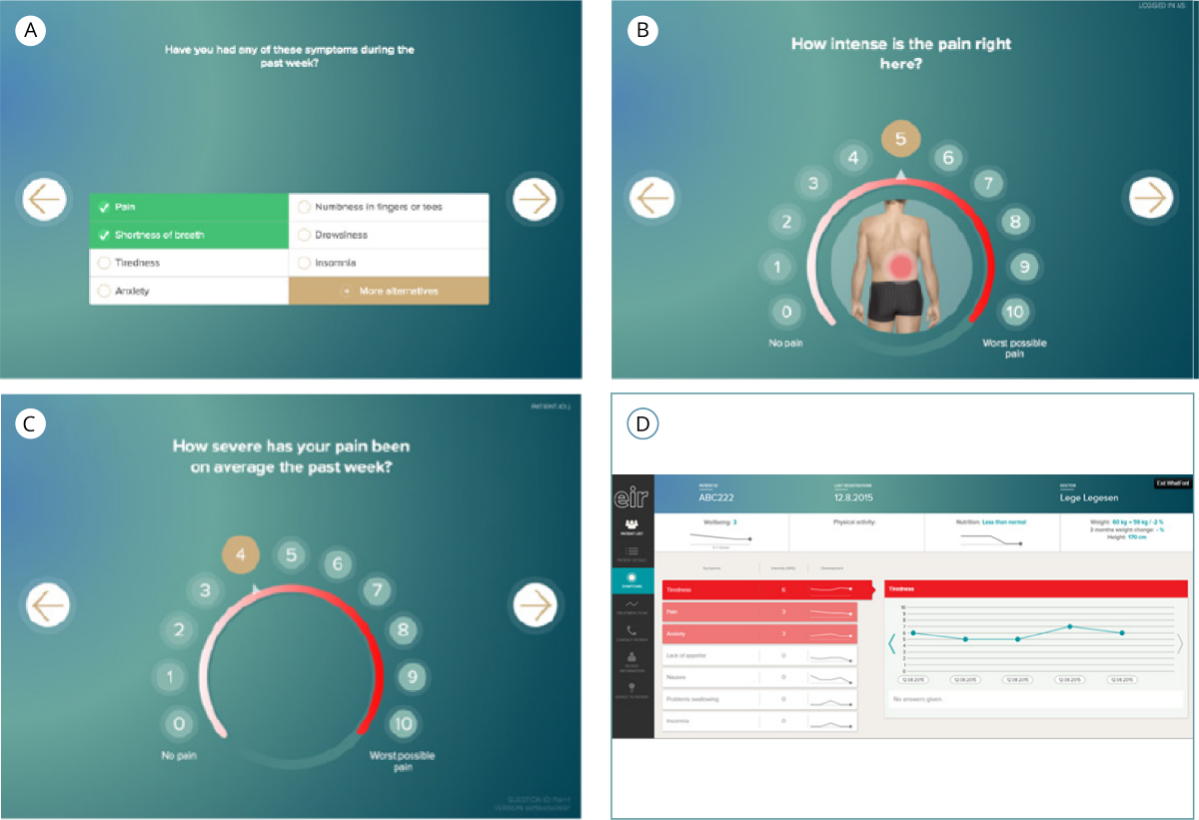
Excerpts from the visual presentation of the conceptual electronic patient-reported outcome measure tool presented to patients in stage 2. (A) Example of symptom selection interface allowing patients to indicate various symptoms; (B) Example of pain mapping including localization and intensity (scale 0‐10), where each marked pain location is assessed individually; (C) Example of symptom rating (here: pain) on a numerical scale from 0 to 10, performed by rotating a virtual wheel; (D) Overview screen for health care professional following completion of the patient’s symptom assessment.

The increased specificity of the guide, combined with targeted follow-up questions, facilitated deeper insights into participants’ perceptions and expectations. These insights were subsequently incorporated into the MyPath-MATRIX project to inform ongoing tool development. Selected excerpts from the visual materials are shown in Figure 2A-D. The complete interview guide, including all questions but excluding the visual content, is available in Multimedia Appendix 2.

All interviews were conducted between February and September 2024 at locations chosen by the participants. One interview was conducted via Zoom (Zoom Communications, Inc), 1 took place at the university premises, and the remaining interviews were held in meeting rooms at outpatient clinics. The first author (TSS), an experienced female cancer nurse and PhD student, conducted all individual interviews. The FGs were moderated by the first author (TSS), with the last author (MHM), an experienced female nurse and qualitative researcher, serving as comoderator. All interviews were audiotaped and transcribed verbatim. To ensure participant anonymity, transcripts were deidentified by the first author (TSS) using unique participant codes. Participants who took part in both stages 1 and 2 were assigned the same participant codes; however, interviews conducted in stage 2 were marked with “S2” to distinguish them. Any potentially identifiable information was removed during the transcription process [25].

As this study used reflexive thematic analysis (RTA), the concept of data saturation was not used to determine sample size or to guide the conclusion of data collection [34]. Instead, data collection was concluded based on the richness and diversity of the material, alongside practical considerations related to participant availability and the overall study design.

### Data Analysis

The data were analyzed as 1 dataset using RTA as described by Braun and Clarke [35,36]. The 6 steps in RTA guided the transition from data engagement to coding and theme development. The process was iterative, moving back and forth between different phases, rather than being a linear progression from start to finish [35].

In the first phase, the first author (TSS) immersed herself in the data by listening to the audiotaped interviews and thoroughly reading and rereading all transcripts. In the second phase, the first author (TSS) coded all data systematically using NVivo (version 14; QRS International). We adopted an inductive, data-driven approach, openly coding the data and identifying meaningful units from the participants’ responses. During this phase, the first and last authors (TSS and MHM) worked closely together, engaging in discussions to review, rename, and create new codes when necessary to better capture the data’s nuances. Data extracts that reflected nuanced aspects of patients’ perspectives and experiences were selected and interpreted to support theme development [35]. As the focus shifted from codes to themes, the analytic process continued with the first author (TSS) clustering multiple codes to generate initial themes and patterns across the entire dataset. Three authors (TSS, MHM, and TL) engaged in in-depth discussions about the created codes, clusters, and initial themes to explore potential shared meanings within the collected data [35]. In the fourth phase, we engaged in an iterative and reflexive process of moving between data extracts, the full dataset, and the developing themes. This process facilitated the identification of patterns of shared meaning across participants, allowing us to construct themes that captured nuanced understandings of symptom management, PCC, and the perceived role of ePROMs in enhancing patient-centered follow-up. Preliminary themes were discussed multiple times, leading us to reorganize and refine them to enhance their richness, coherence, and distinctiveness [35]. Thematic maps were created for each theme, supporting the visualization of the analytic narrative, including connections and boundaries within and between themes, and clarifying the overall thematic structure [35]. In phase 5, themes were refined, defined, and named. The first author (TSS), in close collaboration with all authors, wrote an abstract for each of the 2 final themes, which further developed the analysis. The themes were then reviewed and validated by the entire team to ensure accuracy and coherence. In the final phase of analysis, the first author (TSS) wrote the report, with all authors providing critical input.

### Ethical Considerations

Participants received both written and oral information about the study prior to providing written informed consent [37]. This included details about the study’s purpose, as well as how interview data would be securely stored and anonymized. Participants were also informed about the researchers conducting the interviews, including their professional backgrounds and current institutional affiliations. Anonymization was carried out during the transcription phase, where each participant was deidentified using a unique code. Only the interviewers (TSS and MHM) have access to participants’ identities. To further ensure privacy, all information that could potentially identify individuals, such as departmental affiliations, names of medical personnel, and other contextual details, was anonymized. Transcriptions and audio recordings are securely stored on servers approved for handling sensitive research data. All procedures complied with established ethical standards for research involving human participants [38]. The MyPath-MATRIX project was submitted to the Regional Committees for Medical and Health Research Ethics (REK) but was deemed to fall outside the scope of REK’s mandate (REK Sør-Øst/633827). This study received ethical approval from the institutional Data Protection Officer at both sites. No participants received any compensation for their participation.

## Results

The analysis led to the development of 2 main themes, with a total of 5 subthemes (Table 1).

**Table 1.** Overview of the thematic analysis process, following Braun and Clarke’s reflexive approach [35].^a^

Theme and subtheme	Refined codes (excerpts)
Symptom management in the shadow of disease-centered care	
Patient responsibility and reluctance in symptom reporting	Responsibility for symptom reporting placed on patients Patients do not expect proactive inquiry from clinicians Avoiding symptom disclosure to not “nag”
Perceived quality of care versus unmet needs: a paradox	Absence of structured symptom assessment routines Feeling that essential needs are unmet Open-ended questions are asked but not always sufficient
Side effects—a necessary evil	Side effects are expected and normalized Life after cancer is changed Enduring side effects as necessary for survival
ePROMs:^b^ Bridging holistic care and disease management	
ePROMs—a conceptual facilitator for holistic focus	Heightened awareness of psychosocial and emotional needs ePROMs—A facilitator to ease the burden of remembering ePROMs support holistic self-reporting
Perceived challenges—a barrier to utilization	Perceived value of ePROMs depends on HCP^c^ engagement Difficulty assigning numerical values to symptoms Digital tools must be quick and easy to use

a Overview of theme development based on interviews and 1 focus group, with patients with cancer receiving curative or palliative care. The first column presents the themes, constructed through interpretive engagement with the data, and the subthemes, representing patterns of shared meaning across data extracts. The second column provides excerpts of refined codes, which were developed and adjusted following reviewer’s feedback to better capture the conceptual meaning of the data.

b ePROMs: electronic patient-reported outcome measures.

c HCP: health care professional.

### Theme 1: Symptom Management in the Shadow of Disease-Centered Care

#### Overview

This theme captures how patients in the study made sense of symptom management within a health care context primarily oriented toward disease treatment and progression. Their accounts revealed that regular consultations tended to focus on the biomedical aspects of cancer, while symptom management and broader experiences of living with cancer were often overlooked or marginalized. A shared understanding emerged among patients that it was their own responsibility to redirect the focus of consultations toward symptoms and concerns. This was evident in how they described prioritizing these issues during clinical consultations. We conceptualized this aspect as a subtheme of Theme 1, titled “Patient Responsibility and Reluctance in Symptom Reporting.”

A second subtheme, “Perceived Quality of Care Versus Unmet Needs—A Paradox,” illustrates how patients expressed feeling adequately supported and cared for by HCPs. This sense of being well looked after appeared to reduce their expectations for symptom-focused follow-up. However, their accounts also revealed that this sentiment coexisted with an awareness of unmet needs, particularly regarding aspects of their experience that extended beyond the disease and its treatment.

The third subtheme, “Side Effects—A Necessary Evil,” reflects how patients distinguished between treatment-related side effects and symptoms of the cancer itself. While side effects were acknowledged and often discussed in clinical encounters, symptoms not directly linked to treatment were less frequently addressed. This contributed to a shared understanding that such symptoms were an inevitable part of living with cancer and not something they could expect support for.

#### Subtheme 1: Patient Responsibility and Reluctance in Symptom Reporting

This subtheme reflects a shared understanding among patients that initiating conversations about symptoms was largely perceived as their own responsibility. This sense of responsibility was shaped by the open-ended questions posed by HCPs, which placed the burden on patients to determine what to disclose. Patients described how this dynamic contributed to a reluctance to report symptoms unless they were clearly linked to treatment or directly inquired about.

*You can go into absolutely everything. Both mental and physical. [...] Because it’s quite an open question you get about how things are going. Then it’s really up to me to address [the various symptoms and ailments]*.

[#P7]

Meetings with HCPs, particularly consultations with physicians, were often brief and focused on disease progression as indicated by imaging results or other tests. This focus reinforced the perception that symptoms not directly related to treatment were outside the scope of clinical concern. Moreover, it contributed to a pattern in which patients managed many aspects of their symptom burden independently and sometimes expressed low expectations that HCPs would address these issues.

*But I don’t quite see why they should do that, in a way? [ask questions about all aspects of having cancer]. Because I handle that myself, so it’s fine*.

[#P5]

At the same time, patients appeared to protect the limited time available in consultations, often hesitating to raise concerns they perceived as peripheral to the main clinical agenda.

*We need to be mindful of the physicians’ time- we can handle some symptoms on our own.*

[#P9]

While many patients viewed the physician’s role as primarily focused on diagnosis and treatment, there was a recurring uncertainty about who was responsible for addressing the broader impact of living with cancer, including symptom and side effect management. This uncertainty regarding professional roles was expressed in varied ways. Some patients associated it with not knowing who should maintain a holistic perspective, referred to as “the human aspect,” while others raised such concerns sporadically during clinical encounters, leaving it to HCPs to decide whether to engage with these issues further.

#### Subtheme 2: Perceived Quality of Care Versus Unmet Needs—A Paradox

Patients noted the absence of a systematic focus on symptom management and yet did not interpret this as a shortcoming in their follow-up within specialist health care services. They described feeling well cared for and consistently encountered accommodating and friendly HCPs, which reinforced their confidence in the quality of care. Despite expressing high satisfaction with their treatment and follow-up, patients also conveyed a sense that something was missing, specifically, attention to aspects of their experience not directly related to the disease or its treatment. This created a paradox between the perceived quality of care and their unmet needs.

Despite patients’ overall satisfaction with their care, it became evident that unmet needs persisted. A shared sense of gaps in the management of specific symptoms emerged, suggesting that positive and respectful interactions with HCPs, although appreciated, did not fully compensate for the absence of a structured approach to symptom management.

*The examination of patients who come [to the hospital] must be much more precise. There must be many more questions*.

[#P2]

Patients who had consistent contact with the same HCPs conveyed a shared sense that continuity fostered a sense of security and provided a stable framework throughout their care trajectory.

*Since I’ve had the same doctor here for so long, we’ve talked a lot about many different things. Both about a bit of anxiety and… Many things. But of course, if you constantly, or often, have to deal with a new doctor and such. Because then he doesn’t know you as well and so on*.

[#P1]

In contrast, those who frequently interacted with unfamiliar HCPs expressed that a lack of continuity contributed to feelings of insecurity and made it more difficult to raise personal concerns or discuss broader aspects of their experience.

A shared focus on the physical aspects of having cancer was evident in both participants’ accounts and their interactions with HCPs. The psychological aspect, however, was largely absent from follow-up care, contributing to a sense among patients that something was missing. While the biomedical aspects of care were perceived as thorough and effective, patients noted a lack of structured attention to the broader dimension of care, highlighting a gap in the overall support provided.

*It’s just a CT scan. And a blood test. That’s it. Nothing else. [...] The healthcare system is good at curing the disease. But they are not good at follow-up afterwards*.

[#P10]

#### SubTheme 3: Side Effects—A Necessary Evil

Across patients’ accounts, cancer treatment was consistently associated with considerable side effects that significantly impacted daily life and overall well-being. While these effects were often accepted as an unavoidable part of treatment, they were also described as having a profound impact on QoL, limiting patients’ ability to engage in everyday activities.

*I hardly notice any difference from one day to the next. But I’m terribly short of breath. [...] I try to go for walks and such...but it’s all about resting. Resting, resting, resting. [...] Completely different [life than before]. Now it’s the end of mountain hikes, that’s for sure*.

[#P3]

Side effects were commonly framed by patients as something to be tolerated, an unavoidable consequence of treatment that had to be endured. This perspective reflected a shared understanding of side effects as a necessary evil, closely tied to the belief that enduring them was part of the cost of receiving curative or disease-controlling treatment.

*Of course, when you take chemotherapy over two days, it does something to me. It does. There’s no doubt about it. But it’s something I have to face*.

[#P6]

Patients’ accounts reflected a pronounced emphasis on treatment-related side effects during interactions with HCPs. However, several patients distinguished between side effects and symptoms of the illness itself. This differentiation, reinforced by the clinical focus on side effects, contributed to a shared understanding that symptoms were an inevitable part of living with cancer and, as such, not considered a priority for discussion or intervention.

*It’s not such a big problem, I think, for me. This I can live with. There are things that are much worse. […] it is that I don’t want to bother them. That’s how it is. But I do bring up things that are more serious*.

[#P8-S2]

Among patients who had lived with cancer for an extended period and had encountered others with the disease, a tendency to downplay their own symptoms was evident. This appeared to stem from comparisons with others perceived to be suffering more severely. Such perceptions contributed to a reluctance to raise personal concerns with HCPs, driven by a fear of being a burden or taking up too much of the professionals’ time.

### Theme 2: ePROMs: Bridging Holistic Care and Disease Management

#### Overview

This theme explores how patients made sense of the potential role of an ePROM tool in enhancing patient-centered cancer care. Their accounts highlighted both perceived barriers and facilitators to its use. Patients expressed various expectations and needs they believed the tool could help address, often contrasting these with current clinical practices. A central aspect of this was the belief, shaped by the presentation of the tool’s functionalities, that it could support a more holistic approach to care, extending beyond the physical consequences of cancer. This perspective is developed in the subtheme “ePROMs—A Conceptual Facilitator for Holistic Focus,” reflecting how patients envisioned the tool as a means of promoting attention to the whole person.

Alongside these expectations, patients also identified a range of anticipated challenges related to the tool’s use. These perceived challenges, which stood in contrast to the potential benefits they described, are captured in the subtheme “Perceived Challenges—A Barrier to Utilization.”

#### Subtheme 1: ePROMs—A Conceptual Facilitator for Holistic Focus

Patients anticipated that using an ePROM tool could enhance the focus on all aspects of their being. Several described how completing the assessment in advance might help HCPs see them as whole individuals, rather than solely as patients defined by their disease. This expectation was shaped by the presentation of the tool’s functionalities and reflected a desire for care that acknowledged emotional, psychological, and social dimensions alongside physical health.

*[...] to see the person as well. Not just the disease. That is, the cancer thing. And not just be like on a conveyor belt*.

[#P4]

Simultaneously, patients highlighted the value of completing the ePROM assessment in the peace and quiet of their home prior to consultations. This opportunity for reflection was seen as contributing to a more holistic sense of care. One patient envisioned that this process could help them be more honest about their own situation, as the use of the tool would shift some responsibility onto the HCP to engage with the full scope of their responses:

*It might be better to write it down, and then they [HCPs] can bring it up if they think it’s worth discussing or not. [...] What I might have kept inside. If I can’t say it, then it’s... Yes, check it off and sit at home in peace and quiet to do it [...] It’s easier to check it off, because then they can maybe see if it’s worth discussing or not. They can evaluate it*.

[#P8-S2]

In addition, several patients anticipated that the use of an ePROM tool could help bring greater attention to the psychological dimensions of living with cancer. Their accounts reflected a belief that such a tool could legitimize the view that a cancer diagnosis encompasses more than physical illness alone, thereby encouraging a more holistic approach to care.

*I felt a bit happy when you brought the form [symptom assessment]. Because I felt like, wow. I should have had this from the beginning. Then I could have explained more about what’s inside here [points to head]. Not just that they draw blood from your hand*.

[#P6]

Patients expressed that they did not expect the tool to require substantial training, instead conveying a sense that its use would be intuitive and straightforward. They also emphasized its ease and efficiency, identifying this as a key facilitator in terms of practical implementation and everyday usability.

#### Subtheme 2: Perceived Challenges—A Barrier to Utilization

Among some patients, a pattern of meaning was constructed around concerns related to control and timing in relation to illness focus. Digital tools involving prompts or notifications were described as potentially interfering with the ability to choose when to engage with illness. The possibility that these tools might demand attention at unwanted times was seen as a disruption of personal boundaries, making it more difficult to manage when and how illness became present in everyday life. This perceived loss of control contributed to a broader sense that digital tools could increase the focus on illness in daily life, forming a potential barrier to their use:

*The way it has been until now, it has been a lot of it [focus on the disease]. This [the ePROM tool] must not require too much [time and attention]*.

[#P5]

Patients’ accounts highlighted concerns related to digital literacy and equitable access. Limited familiarity with digital tools was positioned as a potential barrier to engaging with ePROMs. Within this pattern, the importance of voluntary participation was emphasized, alongside concerns that older individuals or others who do not currently use digital technologies might face challenges in accessing and using such tools.

*One should consider training for those who clearly don’t have it [experience with digital tools]. And maybe also without them having to ask for it, because it’s not so easy for many to ask. But to have an offer here that doesn’t require too much*.

[#P7]

Several patients described difficulties in accurately rating individual symptoms using a 0‐10 scale. Uncertainty was expressed about how to position oneself on the scale and what score might be considered significant by HCPs in terms of triggering interventions. Within this pattern, training in how to use such scales was considered essential to prevent them from becoming a barrier to engaging with ePROMs.

Another pattern of meaning emerged regarding the HCP’s approach during consultations. Patients emphasized that if they were to invest time in completing ePROMs beforehand, it was essential that the tool’s purpose was made clear and that it was actively addressed during the consultation. The perceived relevance and integration of the tool into the clinical encounter were seen as key factors influencing its acceptability.

*[...] And a prerequisite is that the doctors use it too. That we don’t sit at home and spend time filling this out, and the doctors don’t read it*.

[#P4-S2]

Patients also highlighted the importance of maintaining physical contact with their HCP. They expressed that the use of such a tool should serve to complement, rather than replace, existing practices.

## Discussion

### Principal Results

This qualitative study aimed to explore the perspectives of patients with cancer on PCC and symptom management, including their experiences with current clinical practice and their views on how ePROMs might enhance patient-centered follow-up. The analysis generated 2 interrelated themes, which together highlight the complex interplay between patients’ lived experiences and their expectations of care.

Theme 1 identified considerable gaps in current clinical practice, particularly concerning symptom management and the delivery of PCC. On one hand, patients described experiencing burdensome symptoms and expressed uncertainty regarding who held responsibility for addressing the broader, more holistic dimensions of cancer care. On the other hand, they reported low expectations that oncologists would, or indeed could, engage with such concerns, perceiving them as beyond the traditional remit of oncology. The challenges and limitations identified in current symptom management appear to directly inform patients’ needs and expectations regarding the use of an ePROM tool. In theme 2, which may be interpreted as a response to the biomedical orientation described in theme 1, patients expressed a desire for a more holistic and patient-centered approach to cancer care. They envisioned ePROMs as a way of bridging this gap by legitimizing and structuring attention to symptoms and aspects of well-being that are frequently overlooked in current practice. The thematic relationship reflects how patients’ experiences of unmet needs influence their perceptions of digital tools designed to enhance follow-up.

While patients generally viewed ePROMs as a promising tool for capturing the lived experience of cancer and facilitating more patient-centered consultations, subtheme 2 of theme 2 revealed a nuanced ambivalence regarding their emotional impact. Patients’ accounts highlighted concerns that regular digital symptom tracking might act as a persistent reminder of illness, thereby intensifying psychological distress and unwanted focus on their condition.

These findings underscore the importance of implementing ePROMs in a way that ensures that the data collected are not only integrated meaningfully into clinical practice but also supported by clearly defined professional roles. The themes reflect a clear desire among patients for a redistribution of responsibility, shifting the burden of symptom management from patients to HCPs. Such a shift not only addresses the emotional and practical challenges patients face but also signals a broader need to reconfigure clinical routines to better accommodate holistic, patient-centered approaches to cancer care.

### Comparison With Prior Work

The primary focus in cancer care remains on treating the disease, curative measures, and life prolongation, often relegating patient-centered approaches to the background [4]. Our findings indicate that this tumor-centered focus is also prevalent among patients. Consequently, symptom management tends to be deprioritized. Among patients with advanced cancer, Monsen et al [39] found that symptoms not directly assessed were reported significantly less frequently than those that were explicitly addressed. Furthermore, time constraints during clinical encounters have been identified as a barrier to raising awareness of non−tumor-centric issues [40], alongside patients’ limited intention to initiate discussions about symptoms with clinicians [41]. This is particularly concerning given the symptom burden experienced by some patients with cancer [42,43] and the potential consequences of unaddressed symptoms [6,44]. In addition, patients with cancer often report a range of unmet needs [45,46]. Thus, these findings highlight the need for a transformation in clinical encounters within cancer care to enhance PCC, particularly through the implementation of systematic symptom management.

Previous research shows that integrating PCC approaches, including systematic symptom management, into clinical care can significantly improve overall care quality and patient outcomes [6,47,48]. This study found that systematic symptom management is neither routinely performed nor expected by patients, despite being an unmet need. Addressing the tumor-centric focus and aligning care with patients’ views on symptom importance may enhance symptom management and support the integration of ePROMs. Simultaneously, this approach could foster PCC and shared decision-making by equipping patients with the necessary competence and understanding to effectively manage their roles [1,49]. Given that symptom burden is a common challenge across many chronic and complex conditions, these insights may also be applicable to other patient populations beyond oncology.

Our findings suggest that patients felt a considerable responsibility for raising symptom-related concerns themselves. The use of open-ended questions by HCPs appeared to reinforce this perception, indicating that broader issues related to the cancer experience were often left for patients to initiate. Prior evidence has shown that the systematic use of (e)PROMs often supports HCPs in the early detection of symptoms, thereby preventing poor outcomes [50-52]. Our findings indicate that the perceived lack of focus on symptoms from HCP reinforces the burden on patients, heightening their sense of responsibility for symptom management and reinforcing a tumor-centered approach. This dynamic may ultimately hinder the implementation of PCC.

Although patients were generally satisfied with their follow-up care, they identified potential for a more holistic and patient-centered approach through the use of ePROMs. The power imbalance between patients and HCPs is a well-documented barrier to PCC [53]. Systematic use of ePROMs has the potential to highlight key concerns [19,22,54]. In addition, this approach may enable patients to shift some of the responsibility for follow-up onto HCPs, thereby supporting more comprehensive and continuous care. In a previous review, Darley et al [55] found that remote symptom management and self-report technology, along with feedback from HCPs, facilitated the feeling of being taken care of and placed responsibility for addressing symptoms and follow-up with HCPs. Other studies have shown that the feelings of patients with cancer being in control and more involved in their own care increase significantly when using ePROMs [14,56]. Implementing ePROMs could significantly reduce the burden on patients by increasing the focus on all symptoms, while simultaneously shifting some of the responsibility for further focus from patients to HCPs.

At the same time, patients acknowledged that ePROMs could introduce challenges not present in current practice. Some expressed concern that prompts to complete symptom reports, such as phone notifications, might draw unwanted attention to their illness in everyday life, particularly when they preferred not to focus on the disease. Similar findings have been reported elsewhere [57]. However, in contrast to this and our findings, the use of telehealth has generally not been found to increase burden for most patients [58]. This may suggest that, while awareness of this potential burden is important, the use of ePROM is unlikely to be perceived as intrusive or overwhelming by the majority of patients, provided it is implemented in meaningful ways that support PCC.

Patients in this study stressed that if they were to allocate time and effort into filling out ePROM, they expected the HCPs to address the issues raised during consultations. Other studies have found that, despite patients filling out PROMs in advance, doctors often did not address the questionnaire in their encounter with the patient [54]. In a previous study, we found that there was a need for a transformation in how patient consultations were conducted to leverage the benefits from using ePROMs [59]. Raising awareness about the need for a cultural shift among both patients and HCPs is crucial to fully realize the potential benefits of systematically using ePROMs in cancer care.

### Future Implications

Despite patients reporting high levels of satisfaction with current cancer care, there remains a need for systematic symptom assessment and follow-up. HCPs must be aware of this and recognize that patients may underreport their symptoms and experiences. A deeper understanding of why patients potentially adopt a tumor-centered focus, and the underlying reasons they may not view symptom management as a priority, could inform the development of strategies to prevent these perceptions from becoming barriers to the successful implementation of ePROMs. Although this study focused on oncology, the emphasis on unmet symptom-related needs and the potential of ePROMs may be transferable to other clinical contexts where symptom burden is similarly underaddressed.

Furthermore, we found that patients did not anticipate needing extensive training to use the tool if they had some degree of digital literacy. However, they found it challenging to quantify their symptoms and score them on a 0-10 scale. This could be a potential barrier to use and may create bias in the assessment. Therefore, implementation in clinical practice, along with comprehensive training, becomes especially important.

Involving patients in qualitative sessions that simultaneously explore current practices can enhance understanding of their preferences and needs, while also providing deeper insight into the requirements for successful implementation of ePROM tools. Moreover, patients in this study distinguished between side effects, viewed as a necessary evil of treatment, and symptoms. Future research should explore this distinction, as it could be crucial for realizing the full benefits of using ePROMs to facilitate a more patient-centered approach to cancer care.

### Strengths and Limitations

Strengths of this study include its qualitative design, which enabled rich, in-depth insight into patients’ perspectives on PCC and symptom management. However, we acknowledge certain limitations, which should be considered when interpreting the results [24]. First, conducting the study within the Norwegian cancer care context may influence the transferability of the findings. Norway’s publicly funded health care system, characterized by universal access and a strong emphasis on digital health integration [60], may differ from systems with limited eHealth infrastructure or more fragmented care. However, while these contextual factors may shape patients’ experiences, the core findings, particularly regarding unmet needs and the perceived value of ePROMs, are likely to be relevant across settings where similar gaps exist.

The relatively small sample size (n=10) may limit the breadth of perspectives captured, although qualitative research prioritizes depth over generalizability [34]. In addition, the use of a specific ePROM prototype may constrain transferability to other digital tools with different interfaces or functionality. Finally, we did not assess patients’ level of digital literacy, which could have influenced their engagement with and perceptions of the tool.

### Conclusions

This study offers important insights into how patients with cancer perceive current approaches to symptom management and PCC, highlighting both satisfaction and areas of unmet need. Patients expressed a sense of personal responsibility in ensuring that their symptoms were addressed, pointing to gaps in follow-up and communication. ePROMs emerged as a promising tool to enhance PCC by amplifying patient voices, enabling more holistic and responsive care, and potentially shifting some of the burden of symptom reporting from patients to HCPs.

Moreover, the findings of this study may have important implications for the design and implementation of eHealth interventions in cancer care. Integrating ePROMs into routine clinical practice could not only enhance the visibility and prioritization of symptom management but also contribute to more equitable care across diverse patient populations. In addition, ePROMs may foster a shared understanding between patients and HCPs regarding the importance of addressing symptoms proactively, thereby supporting a more collaborative and patient-centered approach to follow-up care.

## Supplementary material

10.2196/79144 Multimedia Appendix 1 Interview guide stage 1.

10.2196/79144 Multimedia Appendix 2 Interview guide stage 2.

## Abbreviations:

ePROM: electronic patient-reported outcome measure
FG: focus group
HCP: health care professional
PCC: patient-centered care
PROM: patient-reported outcome measure
QoL: quality of life
RTA: reflexive thematic analysis

## References

[1] Institute of Medicine Committee on Quality of Health Care in America (2001). Crossing the Quality Chasm: A New Health System for the 21st Century.

[2] Rathert C, Wyrwich MD, Boren SA (2013). Patient-centered care and outcomes: a systematic review of the literature. Med Care Res Rev.

[3] Elkefi S, Asan O (2023). The impact of patient-centered care on cancer patients’ QOC, self-efficacy, and trust towards doctors: analysis of a national survey. J Patient Exp.

[4] Kaasa S, Hjermstad MJ, Sjøgren P, European Observatory on Health Systems and Policies (2022). Commercial and social determinants in palliative care. Eurohealth (Lond).

[5] Scherrens AL, Jacobs A, Beernaert K (2024). Integrating patient-centred and tumour-centred cancer care: the EU-MyPath implementation project offers an innovative digital solution with care pathways. Palliat Care Soc Pract.

[6] Balitsky AK, Rayner D, Britto J (2024). Patient-reported outcome measures in cancer care: an updated systematic review and meta-analysis. JAMA Netw Open.

[7] Laugsand EA, Sprangers MAG, Bjordal K, Skorpen F, Kaasa S, Klepstad P (2010). Health care providers underestimate symptom intensities of cancer patients: a multicenter European study. Health Qual Life Outcomes.

[8] Marino D, Baratelli C, Guida G (2020). Impact of adoption of patient-reported outcomes in clinical practice on the accuracy of symptom reporting in medical records of cancer patients. Recenti Prog Med.

[9] Di Maio M, Gallo C, Leighl NB (2015). Symptomatic toxicities experienced during anticancer treatment: agreement between patient and physician reporting in three randomized trials. J Clin Oncol.

[10] Heumann P, Aguado-Barrera ME, Avuzzi B (2023). Comparing symptom reporting by prostate cancer patients and healthcare professionals in the international multicentre REQUITE study. Radiother Oncol.

[11] Zhu S, Dong Y, Li Y (2024). Experiences of patients with cancer using electronic symptom management systems: qualitative systematic review and meta-synthesis. J Med Internet Res.

[12] (2009). Guidance for industry: patient-reported outcome measures: use in medical product development to support labeling claims.

[13] Carfora L, Foley CM, Hagi-Diakou P (2022). Patients’ experiences and perspectives of patient-reported outcome measures in clinical care: a systematic review and qualitative meta-synthesis. PLoS One.

[14] Payne A, Horne A, Bayman N (2023). Patient and clinician-reported experiences of using electronic patient reported outcome measures (ePROMs) as part of routine cancer care. J Patient Rep Outcomes.

[15] Salmani H, Nasiri S, Ahmadi M (2024). The advantages, disadvantages, threats, and opportunities of electronic patient-reported outcome systems in cancer: a systematic review. Digit Health.

[16] Wittich L, Tsatsaronis C, Kuklinski D (2024). Patient-reported outcome measures as an intervention: a comprehensive overview of systematic reviews on the effects of feedback. Value Health.

[17] Chen J, Ou L, Hollis SJ (2013). A systematic review of the impact of routine collection of patient reported outcome measures on patients, providers and health organisations in an oncologic setting. BMC Health Serv Res.

[18] Basch E, Deal AM, Kris MG (2016). Symptom monitoring with patient-reported outcomes during routine cancer treatment: a randomized controlled trial. J Clin Oncol.

[19] Skåre TS, Midtbust MH, Lund JÅ, Kaasa S, Dreyer A (2023). Barriers and facilitators when implementing electronic patient-reported outcome measures at a municipal cancer care unit: a qualitative study. Cancer Nurs.

[20] Glenwright BG, Simmich J, Cottrell M (2023). Facilitators and barriers to implementing electronic patient-reported outcome and experience measures in a health care setting: a systematic review. J Patient Rep Outcomes.

[21] Consolo L, Castellini G, Cilluffo S, Basile I, Lusignani M (2022). Electronic patient-reported outcomes (e-PROMs) in palliative cancer care: a scoping review. J Patient Rep Outcomes.

[22] Meirte J, Hellemans N, Anthonissen M (2020). Benefits and disadvantages of electronic patient-reported outcome measures: systematic review. JMIR Perioper Med.

[23] Howell D, Molloy S, Wilkinson K (2015). Patient-reported outcomes in routine cancer clinical practice: a scoping review of use, impact on health outcomes, and implementation factors. Ann Oncol.

[24] Patton MQ, Angeles L (2015). Qualitative Research and Evaluation Methods: Integrating Theory and Practice.

[25] Braun V, Clarke V (2013). Successful Qualitative Research: A Practical Guide for Beginners.

[26] Kellaghan T, Peterson P, Baker E, McGaw B (2010). International Encyclopedia of Education.

[27] (2024). MyPath-MATRIX. MATRIX.

[28] (2025). MyPath.

[29] Urrizola A, Brkic A, Caraceni A (2025). MyPath: the roadmap to implementing patient-centred care. Acad Oncol.

[30] Krogstad H, Brunelli C, Sand K (2017). Development of EirV3: a computer-based tool for patient-reported outcome measures in cancer. JCO Clin Cancer Inform.

[31] Krogstad H, Sundt-Hansen SM, Hjermstad MJ (2019). Usability testing of EirV3-a computer-based tool for patient-reported outcome measures in cancer. Support Care Cancer.

[32] Malterud K (2025). Kvalitative Forskningsmetoder for Medisin Og Helsefag.

[33] Brinkmann S, Kvale S (2015). InterViews: Learning the Craft of Qualitative Research Interviewing.

[34] Braun V, Clarke V (2021). To saturate or not to saturate? Questioning data saturation as a useful concept for thematic analysis and sample-size rationales. Qual Res Sport Exerc Health.

[35] Braun V, Clarke V (2022). Thematic Analysis: A Practical Guide.

[36] Braun V (2021). Should I not use TA? Comparing reflexive thematic analysis and other pattern-based qualitative analytic approaches. Couns Psychother Res.

[37] Creswell JW, Creswell JD (2018). Research Design: Qualitative, Quantitative & Mixed Methods Approaches.

[38] (2024). WMA Declaration of Helsinki—ethical principles for medical research involving human participants. World Medical Association.

[39] Monsen RE, Lerdal A, Nordgarden H, Gay CL, Herlofson BB (2024). A comparison of the prevalence of dry mouth and other symptoms using two different versions of the Edmonton Symptom Assessment System on an inpatient palliative care unit. BMC Palliat Care.

[40] Pollak KI, Arnold RM, Jeffreys AS (2007). Oncologist communication about emotion during visits with patients with advanced cancer. J Clin Oncol.

[41] Penalba V, Deshields TL, Klinkenberg D (2019). Gaps in communication between cancer patients and healthcare providers: symptom distress and patients’ intentions to disclose. Support Care Cancer.

[42] Bubis LD, Davis L, Mahar A (2018). Symptom burden in the first year after cancer diagnosis: an analysis of patient-reported outcomes. J Clin Oncol.

[43] Bubis LD, Davis LE, Canaj H (2020). Patient-reported symptom severity among 22,650 cancer outpatients in the last six months of life. J Pain Symptom Manage.

[44] Basch E, Deal AM, Dueck AC (2017). Overall survival results of a trial assessing patient-reported outcomes for symptom monitoring during routine cancer treatment. JAMA.

[45] Harrison JD, Young JM, Price MA, Butow PN, Solomon MJ (2009). What are the unmet supportive care needs of people with cancer? A systematic review. Support Care Cancer.

[46] Wang T, Molassiotis A, Chung BPM, Tan JY (2018). Unmet care needs of advanced cancer patients and their informal caregivers: a systematic review. BMC Palliat Care.

[47] Kaasa S, Loge JH, Aapro M (2018). Integration of oncology and palliative care: a Lancet Oncology Commission. Lancet Oncol.

[48] Howell D, Fitch M, Bakker D (2013). Core domains for a person-focused outcome measurement system in cancer (PROMS-Cancer Core) for routine care: a scoping review and Canadian Delphi Consensus. Value Health.

[49] Grol R (2013). Improving Patient Care: The Implementation of Change in Health Care.

[50] Basch E, Hudson K, Rocque G (2023). Implementation of electronic patient-reported outcomes for symptom monitoring during cancer treatment: the importance of getting it right. J Comp Eff Res.

[51] Basch E, Schrag D, Henson S (2022). Effect of electronic symptom monitoring on patient-reported outcomes among patients with metastatic cancer: a randomized clinical trial. JAMA.

[52] Di Maio M, Basch E, Denis F (2022). The role of patient-reported outcome measures in the continuum of cancer clinical care: ESMO Clinical Practice Guideline. Ann Oncol.

[53] Joseph-Williams N, Elwyn G, Edwards A (2014). Knowledge is not power for patients: a systematic review and thematic synthesis of patient-reported barriers and facilitators to shared decision making. Patient Educ Couns.

[54] Lombi L, Alfieri S, Brunelli C (2023). “Why should I fill out this questionnaire?” A qualitative study of cancer patients’ perspectives on the integration of e-PROMs in routine clinical care. Eur J Oncol Nurs.

[55] Darley A, Coughlan B, Furlong E (2021). People with cancer and their family caregivers’ personal experience of using supportive eHealth technology: a narrative review. Eur J Oncol Nurs.

[56] Basch E, Iasonos A, Barz A (2007). Long-term toxicity monitoring via electronic patient-reported outcomes in patients receiving chemotherapy. J Clin Oncol.

[57] Steindal SA, Nes AAG, Godskesen TE (2023). Advantages and challenges of using telehealth for home-based palliative care: systematic mixed studies review. J Med Internet Res.

[58] Steindal SA, Nes AAG, Godskesen TE (2020). Patients’ experiences of telehealth in palliative home care: scoping review. J Med Internet Res.

[59] Skåre TS, Lundeby T, Lund JÅ, Hjermstad MJ, Midtbust MH (2025). First-time use of electronic patient-reported outcome measures in a cluster randomized trial: a qualitative study. J Patient Rep Outcomes.

[60] Saunes IS, Karanikolos M, Sagan A (2020). Norway: health system review. Health Syst Transit.

[61] Jennifer S (2024). Can JAMA network authors use generative artificial intelligence to create content?. AMA Style Insider.

