# Supplementary material for: Exploring Perspectives of Patients With Cancer on Implementing Electronic Patient-Reported Outcome Measures to Enhance Patient-Centered Care: Qualitative Study
Source: JMIR Cancer. 2025 Nov 25;11:e79144. doi: 10.2196/79144 (PMC12646547; doi:10.2196/79144)
Supplement: Multimedia Appendix 1 [file cancer-v11-e79144-s001.docx]

Multimedia Appendix 1. Interview guide Stage 1

| Background | - Could you describe how often and for how long you are at the hospital - Who is your main point of contact in the healthcare system when you need help or advice |
| --- | --- |
| Experiences associated with PCC | - Could you describe how you experience PCC in your interactions with HCPs - How does the topics related to PCC usually come up during consultation? - How do you feel these issues are followed up? - What do you expect from your oncologist/other relevant HCPs when it comes to following up on these matters? - What do you expect your oncologist/other relevant HCPs to do if you raise these concerns - What are your expectations for the consultation? - What is important for you to talk about? - How do you experience getting in touch with the healthcare system when you need to? |
| Experiences associated with the collection of symptoms and ailments | - Could you describe how you perceive the current focus on symptoms and ailments - Have you ever filled out symptom assessment forms, either digitally or on paper? - If yes: How was your experience reporting symptoms this way? - Could you describe your expectations regarding the follow-up of symptoms and ailments |
| Experiences associated with the use of digital solutions in relation to health | - Are you currently using any digital solutions - Expectations for a digital tool for symptom assessment - If available, would you consider using a digital tool to assess your current situation? Why/why not? |
| Summary | - Is there any additional topic you would like to discuss that we have not yet addressed |
