# Supplementary material for: Exploring Perspectives of Patients With Cancer on Implementing Electronic Patient-Reported Outcome Measures to Enhance Patient-Centered Care: Qualitative Study
Source: JMIR Cancer. 2025 Nov 25;11:e79144. doi: 10.2196/79144 (PMC12646547; doi:10.2196/79144)
Supplement: Multimedia Appendix 2 [file cancer-v11-e79144-s002.docx]

Multimedia Appendix 2. Interview guide Stage 2

| Prior to the consultation | - Could you describe how you currently prepare for a hospital consultation? - When would it feel most appropriate for you to complete a digital self-assessment? - Would you find it helpful to receive a reminder? - Which digital platform or device would be most convenient for you to use? - What would you expect/prefer to happen after submitting your responses? - Would receiving general advice or guidance through the tool be helpful? - The responses from the digital assessment will first be addressed during the consultation itself. What is your thought on this approach? |
| --- | --- |
| During the consultation | - What are your thoughts on the idea that the consultation will be based on the information you submitted in advance? - Do you anticipate that this approach might change the nature of the clinical consultation compared to how it is currently conducted? - If you report a high number of symptoms, it might not be possible to address all of them during the consultation. What are your thoughts on that? |
| Visual presentation of the preliminary content and functionalities | - Are the symptoms listed relevant? Anything missing from the list? - What are your thoughts on what you see on the screen here? - How do you interpret what is being asked of you? - How do you experience the task of rating a symptom on a scale from 0 to 10? - Do you think you might need help using this tool? If yes, what kind of help would be useful? - Do you think you would need any support or training to use it? If yes, what kind? |
| Summary | - Do you believe that an ePROM tool would be of value to you? - What would be necessary for you to consider using such a tool? (Barriers/Facilitators) - Is there anything else you feel is important to highlight in relation to this topic? |
